# A novel 1,4-naphthoquinone-derived compound induces apoptotic cell death in breast cancer cells

**DOI:** 10.3906/biy-1901-19

**Published:** 2019-08-05

**Authors:** Didem KARAKAŞ, Remzi Okan AKAR, Zeliha GÖKMEN, Nahide Gülşah DENİZ, Engin ULUKAYA

**Affiliations:** 1 Department of Medical Biochemistry, Faculty of Medical School, İstinye University, İstanbul, Turkey; 2 Department of Molecular Biology and Genetics, Faculty of Science and Literature, İstinye University, İstanbul, Turkey; 3 Department of Cancer Biology and Pharmacology, Institute of Medical Sciences, İstinye University, İstanbul, Turkey; 4 Division of Organic Chemistry, Department of Chemistry, Faculty of Engineering, İstanbul University-Cerrahpaşa, İstanbul, Turkey

**Keywords:** Quinones, 1, 4-naphthoquinones, cytotoxicity, apoptosis, triple-negative breast cancer, anticancer agent

## Abstract

Breast cancer is the most-diagnosed cancer type among women. The triple-negative subtype is an especially aggressive type of breast cancer. Although chemotherapy is almost the only option for the treatment of triple-negative breast cancer (TNBC), currently used chemotherapeutics are not effective enough, considering the poor survival rate of patients. Therefore, novel compounds need to be developed to improve survival rates. It has been known that quinonic compounds, which are found in nature, have antibacterial, antifungal, and antitumorigenic properties. Naphthoquinones are members of the quinone family and are widely used in research due to their promising properties. In this study, we evaluated the cytotoxic activity of a novel naphthoquinone-derived compound (1,4-naphthoquinone (1,4-NQ)) against two different breast cancer cells: a hormone-responsive cell line (MCF-7) and a triple-negative cell line (MDA-MB-231). As a result, 1,4-NQ decreased cell viability in both tested cell lines in a dose-dependent manner. Increased apoptotic markers (presence of pyknotic nuclei, annexin-V positivity, caspase 3/7 activity, and decreased mitochondrial membrane potential) and DNA damage were especially observed in MDA-MB-231 cells after treatment with the compound. Considering the promising cytotoxic effect of the compound, 1,4-NQ needs further evaluation as a potential candidate for the treatment of TNBC.

## 1. Introduction

**Figure 1:**
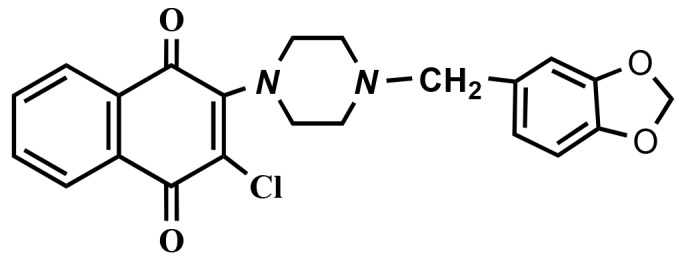
Structure of compound.

Breast cancer is the second most commonly diagnosed cancer after lung cancer for both sexes combined. Breast cancer is also the leading cause of cancer death among women. It is estimated that there were 2.1 million newly diagnosed female breast cancer cases in 2018. Despite recent progress in early detection and new therapeutic strategies, the rate of incidence is increasing worldwide (Bray et al., 2018).Triple-negative breast cancer (TNBC), a subtype of breast cancer, is characterized by the lack of estrogen, progesterone, and human epidermal receptors (Brenton et al., 2005). TNBC is a biologically aggressive type of breast cancer with a poor survival rate (Foulkes et al., 2010). Although chemotherapy is accepted as the standard treatment for TNBC, prognosis remains poor (Yao et al., 2017). Therefore, novel compounds are still being synthesized and investigated in the fight against breast cancer.Quinones widely occur in living cells. Thus, synthetic and natural quinones have been the subject of several studies, especially regarding their anticancer activities (Dolan et al., 1998; Monks et al., 2002; Verma et al., 2006; Hillard et al., 2008; da Silva et al., 2009; Prachayasittikul et al., 2014). In the quinone family, 1,4-naphthoquinones are the most important and widely used group in research (da Silva et al., 2002; Ravelo et al., 2004; Tandon et al., 2004). Many clinically important antitumorigenic drugs contain a quinone nucleus (such as anthracyclines, mitoxantrone, and saintopin) and these drugs effectively inhibit DNA topoisomerases (Foye, 1995). In addition, quinone analogues can produce superoxide radicals, due to inducing the formation of semiquinone radicals. Both the superoxide and semiquinone radical anions of naphthoquinone analogues can generate the hydroxyl radical, which is known to cause breaks in DNA strands (Tewey et al., 1984). Considering these effects of naphthoquinones, it has been suggested that these compounds might exhibit cytotoxic activities against cancer cells. Consistent with these predictions, several studies have shown that the naphthoquinone analogues have anticancer effects (Dolan et al., 1998; Tandon et al., 2004; Verma et al., 2006; Prachayasittikul et al., 2014; Pingaew et al., 2015; Ghosh et al., 2018). Our previous study demonstrated the synthesis of compound 2-[1-piperonylpiperazin-1-yl]-3-chloro-1,4-naphthoquinone and its cytotoxic effects against lung, breast, prostate, and colon cancer cell lines (Deniz et al., 2015) (Figure  1).In light of this information, in this study we investigate the cytotoxic and apoptotic effects of the compound against two different breast cancer cell lines MCF-7 (ER+, PR+, HER2) and MDA-MB-231 (aggressive and triple-negative). As a result, the compound exhibits cytotoxic activities against both cell lines in a dose-dependent manner. In addition, mitochondria-mediated apoptotic cell death was detected in triple-negative MDA-MB-231 cells, indicating the potential importance of the compound.

## 2. Materials and methods

### 2.1. Chemicals and cell culture

The synthesis and characterization methods of the compound were published by Deniz et al. (2015). A solution of 25 mM was prepared in dimethyl sulfoxide (DMSO) as a stock solution. Further dilutions were made in a culture medium containing 0.1% DMSO. Human breast cancer cell lines, MCF-7 and MDA-MB-231, were cultured in Roswell Park Memorial Institute (RPMI)-1640 medium supplemented with penicillin G (100 U/mL), streptomycin (100 µg/mL), L-glutamine (Lonza, Switzerland), and 10% fetal bovine serum (GIBCO, USA) at 37 °C in a humidified atmosphere containing 5% CO2. 

### 2.2. Sulforhodamine B (SRB) cell viability assay

Cytotoxic activities of the compound were examined using the SRB cell viability assay. Briefly, MCF-7 and MDA-MB-231 cells were seeded at a density of 5 × 103 cells per well in 96-well culture plates and then treated with different concentrations of the compound (in the range of 1.56-50 µM) for 12, 24, and 48 h.Following the indicated treatment periods, 50 µL of 50% trichloroacetic acid (TCA, T6399, Sigma Aldrich, USA) was added and fixation was allowed to proceed for 1 h at 4 **°**C. The supernatants were then discarded and plates were washed with deionized water five times. The cells were incubated with 50 µL of SRB solution (0.4% in 1% acetic acid, sc-253615, Santa Cruz Biotechnology, USA) for 30 min at room temperature to allow the binding of the SRB dye to the basic amino acid residues. Next, the unbound SRB dyes were washed out with 1% acetic acid (695092-2.5L, Sigma Aldrich, USA) and air-dried. Bound SRB was solubilized with 150 µL of Tris base (10 mM, pH 10, 10708976001, Sigma Aldrich, USA) and then plates were shaken for 10 min at 150 rpm. The absorbance values were measured using a spectrophotometer at 564 nm.

### 2.3. Fluorescent staining for the determination of cell death mode

To determine cell death mode based on nuclear morphology and membrane integrity, two different fluorescent dyes (Hoechst 33342 and propidium iodide, Sigma Aldrich, USA) were used. In order to carry out fluorescent staining, MDA-MB-231 cells were seeded at a density of 5 × 103 cells per well in 96-well plates. After 24 h, the cells were treated with 12.5 µM of the compound for 12 and 24 h. At the end of the treatment period, the cells were stained with Hoechst 33342 (5 µg/mL) and propidium iodide (1 µg/mL) and incubated for 20 min at room temperature. Following incubation with dyes, cells were monitored via fluorescence microscopy.

### 2.4. Investigation of caspase 3/7 activity and annexin-V staining

The Muse Cell Analyzer was used to perform annexin-V and caspase 3/7 activity assays. Early/late apoptotic cell death or necrosis was detected using the Annexin-V/Dead Cell Kit (MCH100105, Millipore). The Caspase-3/7 Kit was chosen to investigate the activity of caspase 3 and 7, which are activated in apoptotic cell death (MCH100108, Millipore).To perform the assays, MDA-MB-231 cells were seeded at a density of 2 × 105 cells per well of 6-well plates. A 12.5 µM concentration of the compound was applied 24 h later, and the cells were incubated for an additional 12 and 24 h. At the end of the treatment period, the cells were trypsinized and analyzed for the detection of annexin-V stained cells and caspase 3/7 activity according to the manufacturer’s protocol (Millipore, Hayward, CA, USA).

### 2.5. Evaluation of Bcl-2 phosphorylation and JC-1 staining

For flow cytometric analysis of the changes in the Bcl-2 phosphorylation, MDA-MB-231 cells were seeded and treated with the compound for 12 h. Then the cells were analyzed using the Muse® Bcl-2 Activation Dual Detection Kit | MCH200105 (Millipore) according to the manufacturer’s instructions.To perform JC-1 staining, the cells were seeded at a density of 2.5 × 103 cells per well in a 96-well culture plate and treated with 12.5 µM compound for 12 and 24 h. After treatment periods, the JC-1 staining solution (Cayman Chemical Company, USA) was prepared by diluting the reagent 1:10 in the culture medium and 20 µL of this solution was added to each well of the plate. Following this process, the cells were incubated at 37 **°**C in a humidified atmosphere containing 5% CO2 for 20 min and then the stained cells were analyzed via fluorescence microscopy.

### 2.6. Determination of DNA damage (γH2AX staining)

Histone H2A.X resides downstream of the DNA damage kinase signaling cascade and phosphorylation of H2A.X at serine 139 is known as an indicator of DNA damage. To measure whether the compound induces DNA damage or not, MDA-MB-231 cells were seeded and treated with the compound as described in Section 2.4. Then the cells were collected, fixed, and permeabilized with Muse Fixation Buffer and Permeabilization Buffer, respectively (Muse H2AX Activation Dual Detection (kit MCH200101), Millipore, Darmstadt, Germany). Cells were incubated with a mixture of 2.5 µL of antiphospho-histone H2A.X and 2.5 µL of antihistone H2A.X, PECy5 for 30 min in the dark at room temperature. Then the cells were resuspended and analyzed using the Muse H2AX Activation Dual Detection kit, according to the manufacturer’s instructions.

## 3. Results

### 3.1. The compound caused a striking decrease in the viability of breast cancer cell lines

**Figure 2:**
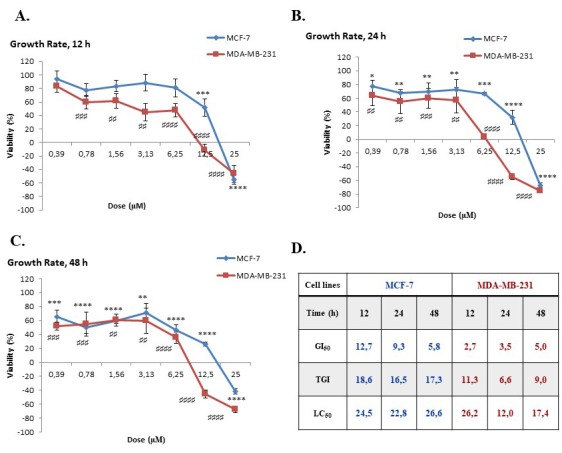
Antigrowth effect of compound against breast cancer cells. Growth rate curves were obtained by the SRB cell viability assay****(A, B, C). GI50, LC50, and TGI values were calculated as explained in the text. GI50, 50% growth inhibition; TGI, total growth inhibition; LC50, lethal dose 50% (D). Statistical significance between different concentrations of the compound and a negative control was compared by analysis of variance (ANOVA) with Dunnett’s multiple comparison posttest.

MCF-7 and MDA-MB-231 cells were treated with the compound as indicated in Section 2.2. After the treatment periods (12, 24, and 48 h), the cytotoxic/cytostatic activities of the compound were evaluated using the SRB cell viability assay. In light of the SRB results, it was found that the compound decreased cell viability in a dose-dependent manner in both MCF-7 and MDA-MB-231 cells. The highest cytotoxic activity of the compound was observed 24 and 48 h after treatment. While the cytotoxicity was slightly lower in the 12 h treatment group, the cytotoxic activity of the compound increased relatively late in treatment. Although the compound exhibited cytotoxic effects on both cell lines, MDA-MB-231 cells were found more sensitive than MCF-7 cells (Figures 2A-2C). Additional growth parameters (GI50, TGI, and LC50 values) were calculated using the formula described in our group’s recent study (Ulukaya et al., 2014). Briefly, GI50 indicates the antiproliferative activity, TGI indicates the cytostatic effects, and LC50 indicates the cytotoxic effects of the tested compound. GI50, TGI, and LC50 values were also found lower in MDA-MB-231 cells when compared to MCF-7 cells (Figure  2D).

### 3.2. Exposure to the compound induces mitochondria-mediated apoptosis in MDA-MB-231 cells

**Figure 3:**
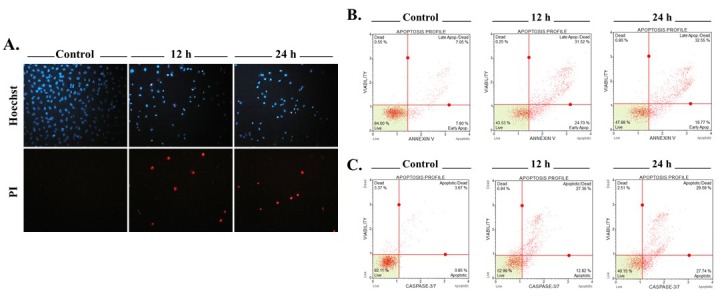
Examination of cell death mode in MDA-MB-231 cells. Cells were stained with Hoechst 33342 (blue) and propidium iodide (red) after being treated with the compound (12.5 μM) for 12 and 24 h (A). Flow cytometric analysis of annexin-V staining (B) and caspase 3/7 activity (C) after treatment with the compound (12.5 μM) for 12 and 24 h. Four populations detected in each cell line by fluorometric separation: nonapoptotic live (lower left), nonapoptotic dead (upper left), early apoptotic (lower right), and late apoptotic (upper right).

**Figure 4:**
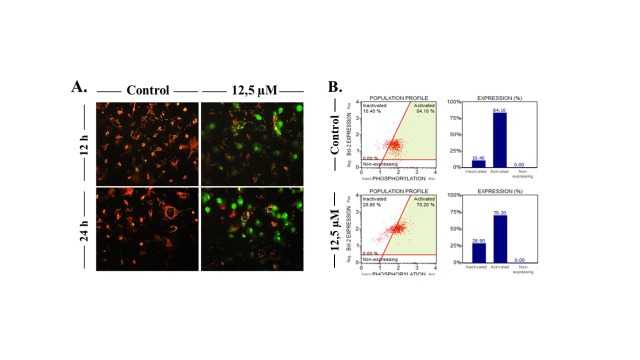
Determination of mitochondrial changes using fluorescent JC-1 staining and flow cytometry. Alterations in the Bcl-2 phosphorylation (A) and Bcl-2 activation (B) in compound-treated MDA-MB-231 cells.

On the basis of the results of the SRB assay, the concentration of the compound for further studies was chosen to be 12.5 µM for MDA-MB-231 cells. To further investigate whether the growth-inhibiting effect of the compound was caused by apoptosis or not, cell death mode was assessed with fluorescent stains on the basis of nuclear morphology and cell membrane integrity. The apoptosis-inducing effect of the compound was shown with the presence of pyknotic nuclei in MDA-MB-231 cells. In addition, secondary necrosis (late apoptosis) was evidenced by pyknotic nuclei with propidium iodide (PI) positivity (Figure  3A).Caspase 3/7 activity and annexin-V positivity in cells were analyzed using the Muse Cell Analyzer for further investigation. After treatment with the 12.5 µM concentration of the compound for 12 and 24 h, increased caspase 3/7 activity and annexin-V positivity were observed in cells (Figures 3B and 3C).The compound also induced changes in the mitochondrial membrane potential (ΔΨm) as shown by JC-1 staining; the green emission is an indication of ΔΨm loss as shown in Figure  4A. Flow cytometric Bcl-2 analysis was also performed to confirm this observation. Since Bcl-2 proteins regulate mitochondria-mediated apoptosis, the serine 70 phosphorylation of Bcl-2 (inactivated Bcl-2) was investigated by the Muse Cell Analyzer. A dramatic increase was found in the percentage of inactivated Bcl-2 in a time-dependent manner (Figure  4B).

### 3.3. DNA damage is induced by the compound in MDA-MB-231 cells

**Figure 5:**
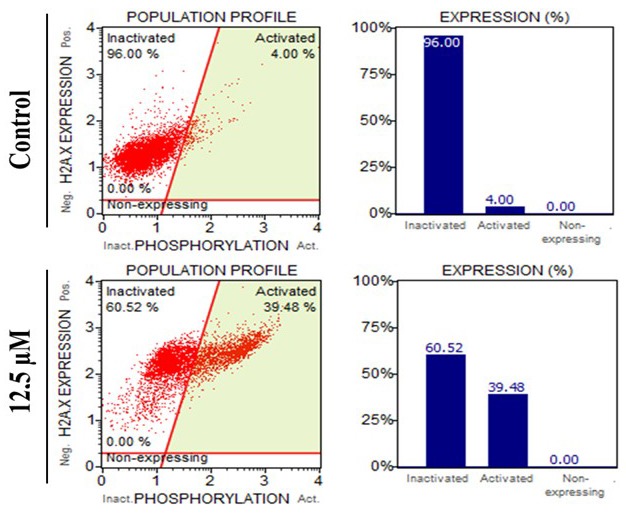
Analysis of DNA damage.****Flow cytometric analysis of (γ-H2AX activation in MDA-MB-231 cells after treatment with 12.5 µM of the compound for 12 h.

To detect DNA double-strand breaks, a gamma H2AX (γ-H2AX) assay was performed by using flow cytometry after the cells were treated with the compound (12.5 µM) for 12 h. The results of the γ-H2AX assay indicated that the compound induced an increase of γ-H2AX levels (which in graphs is referred to as “activated”) in MDA-MB-231 cells (Figure  5).****

## 4. Discussion

TNBC, which is identified by the lack of expression of estrogen receptor (ER), progesterone receptor (PR), and human epidermal growth factor receptor 2 (HER2), is the most aggressive subtype of breast cancer (Guerneri et al., 2013; Zaharia et al., 2013; Schmadeka et al., 2014). Despite improving the treatment success rate for hormone-responsive subtypes of breast cancer (ER+ and HER+), chemotherapy still remains the only treatment option for TNBC. Therefore, novel compounds need to be synthesized and tested to overcome the aggressiveness of TNBC.Nearly four decades ago, studies conducted by the National Cancer Institute (NCI-USA) showed that synthetic and natural quinones possess anticancer activities (Driscoll, 1974). These 1,4-NQ-based compounds have cytotoxic effects against several types of cancer, including breast cancer cells (Verma et al., 2006; da Silva et al., 2009). The cytotoxic activity of compound 2-[1-piperonylpiperazine-1-yl]-3-chloro-1,4-naphthoquinone was previously investigated in several cancer cell lines including MCF-7 cells and promising results were published (Deniz et al., 2015). Therefore, in this study, in addition to MCF-7 cells, we aimed to investigate the potential cytotoxic activity of the compound in a TNBC subtype cell line, MDA-MB-231. As shown in Figure  2the compound decreased cell viability in both MCF-7 and MDA-MB-231 cells in a time- and dose-dependent manner. MDA-MB-231 cells were found more sensitive to the compound compared to MCF-7 cells. In correlation with our result, Pilco-Ferreto and Calaf (2016) showed that MDA-MB-231 breast cancer cells were more sensitive to one of the quinone-based drugs, doxorubicin, compared to MCF-7 breast cancer cells. After our cytotoxicity results, we carried out advanced analyses to elucidate the compound’s mechanism of action.The cell death mode was found to be apoptosis in MDA-MB-231 cells. To evaluate the mode of cell death, fluorescent dye-stained cells (Hoechst 33342 and PI) were analyzed based on nuclear morphology and plasma membrane integrity. While Hoechst 33342 dye stains both viable and dead cells, PI can stain only the cells with a damaged membrane (primary necrotic or secondary necrotic - late stage of apoptosis) (Mazzini et al., 2003). Our results showed that treatment with the compound caused pyknotic nuclei and PI positivity even in early hours of treatment (12 h), indicating late apoptotic cell death (Figure  3A). One of the hallmarks of apoptosis is the translocation of phosphatidylserine (PS) from the inner to outer plasma membrane (Rysavy et al., 2014). When cells undergo apoptosis caspase 3 and 7 are activated via cleavage. Active caspases cleave Xkr8 (XK-related protein 8), which acts as a lipid scramblase resulting in PS exposure to the outer plasma membrane (Mariño et al., 2013; Segawa et al., 2015). Thus, we examined the mode of cell death by flow cytometric analysis based on PS translocation (annexin-V staining) and caspase 3/7 activity. We demonstrated that the compound significantly increased PS translocation and caspase 3/7 activity in MBA-MB-231 cells (Figures  3B and  3C).Quinones can be transformed to semiquinone radicals by the cytochrome P450 reductase enzyme. The semiquinones can be oxidized into quinones under normal oxygen levels. In this process, O2 is reduced, resulting in the production of superoxide radical anions (O2-). Both the superoxide and semiquinone radical anions can generate the hydroxyl radical, which is known to cause breaks in DNA strands (Tewey et al., 1984; Wellington and Kevin, 2015). DNA-damaging agents may activate mitochondrial damage pathways, leading to cell death via apoptosis (Kaina, 2003). Cell death is mostly related to the mitochondrial pathway of apoptosis in a caspase-dependent manner in vertebrates (Green et al., 2004; Ouyang et al., 2012). Our findings demonstrated that exposure of cells to the compound significantly induced loss of mitochondria membrane permeability in MDA-MB-231 cells, suggesting that mitochondria-mediated apoptosis was induced (Figure  4A). Moreover, as shown in Figure  4B, the compound decreased the activity of Bcl-2 in a time-dependent manner. Based on these findings, we suggest that MDA-MB-231 cells undergo mitochondria-dependent apoptosis after treatment with the compound.Quinone-based drugs (such as doxorubicin, mitoxantrone, streptonigrin, and mitomycin C) have been widely used in the treatment of breast cancer. These drugs’ mechanism of action is based on DNA-targeting (DNA intercalation, crosslinking, strand breaks, etc.) (Wellington and Kevin, 2015). As we previously stated above, the superoxide and semiquinone radical anions can also cause DNA strand breaks. H2AX phosphorylation at serine 139 is known as a marker for DNA damage (Sharma et al., 2012). Our results showed that the compound causes an increase in H2AX phosphorylation (DNA damage) in MDA-MB-231 cells. Taken together, these findings indicate that the compound promotes mitochondrial apoptosis in TNBC cells. Considering the challenges in the treatment of TNBC, further investigation is needed to evaluate the detailed mechanisms of action.

## Acknowledgment

The authors would like to express their gratitude to the Scientific Research Projects Coordination Unit of İstanbul University Cerrahpaşa for financial support (Project Number: FBA-2018-28971 and Project Number: 56964).
